# Painless Nodular Cheek Skin Swelling: A Clinical Challenge

**DOI:** 10.7759/cureus.65990

**Published:** 2024-08-02

**Authors:** Deepak Saharan, Varchasvi Meena, Anugrah Mittal, Dilkhush Meena, Lalit K Choudhary

**Affiliations:** 1 Otolaryngology and Head and Neck Surgery, Government Medical College, Kota, Kota, IND

**Keywords:** modified european salivary gland society classification system, superficial parotidectomy, infiltrating basal cell carcinoma, basal cell adenocarcinoma salivary gland, basaloid neoplasms

## Abstract

Basaloid neoplasms of the head and neck region pose a specific challenge both for clinicians and pathologists. It is a diverse group of neoplasms that include benign as well as malignant entities. These neoplasms can arise from various head and neck subsites such as skin, salivary gland, and sinonasal tract. Cytological diagnosis of these tumors is extremely difficult due to morphological overlap with other biphasic tumors and within the basaloid group itself. Here, we are presenting a case of basaloid neoplasm which turned out to be a basal cell adenocarcinoma of the left parotid gland on postoperative histopathological examination.

## Introduction

Basaloid neoplasms of the head and neck region pose a specific challenge both for clinicians and pathologists. It is a diverse group of neoplasms that includes benign as well as malignant entities. The basaloid neoplasm term is used for tumors containing cells showing coarse chromatin in round nuclei and sparse cytoplasm. These cells resemble cells of the epithelial basal layer, hence the term “basaloid.” These can affect various subsites of the head and neck region, such as the skin, salivary gland, and sinonasal tract. The diagnosis of basaloid neoplasm is quite difficult in preoperative cytology [1,2]. Here, we are presenting a case of basaloid neoplasm that was misinterpreted in cytology and clinically.

## Case presentation

**Figure 1:**
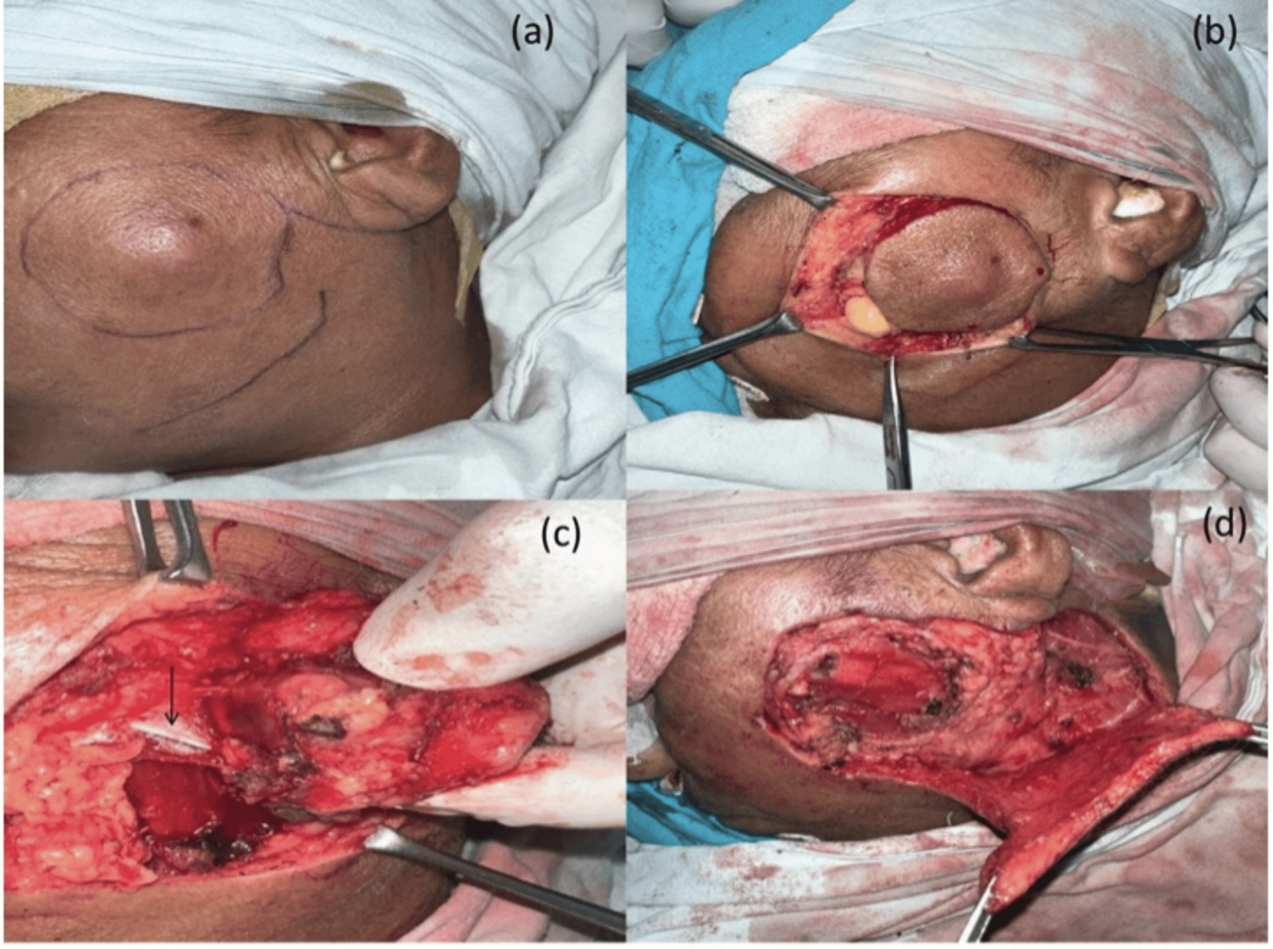
(a) Nodular cheek skin lesion with marking for excision and flap elevation; (b) lesion excised with 1 cm margin all around; (c) buccal branch (black arrow) was infiltrated by the tumor hence sacrificed; (d) elevation of cervicofacial rotation flap

A 67-year-old lady presented to the otorhinolaryngology department with the chief complaint of nodular swelling in her left cheek for five to six months which was painless and gradually increasing in size and no other complaints. On local examination, she was found to have an indurated nodular swelling in her left cheek region approximately 3 cm below the zygoma and approximately 5 cm anterior to the tragus. The swelling was approximately 2 x 2 cm in size, firm in consistency, involving cheek skin, non-discharging, non-tender to touch, having ill-defined margins, and restricted mobility (Figure 1a). No lymph node was palpable on neck examination.

**Figure 2:**
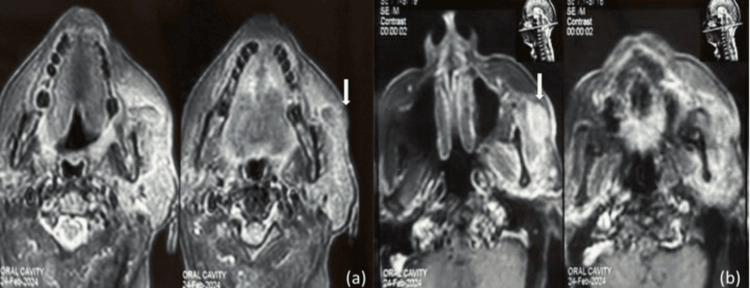
(a) T2W MRI axial section showing heterogeneous iso to hyperintense tumor with loss of subcutaneous fat plane (white block arrow); (b) T1W gadolinium MRI axial section showing hyperintense tumor (white block arrow)

The patient was thoroughly investigated and cytology showed tightly packed cell aggregates having palisading nuclei along the edges of aggregates. Cells are showing enlarged nuclei, a high N:C ratio, irregular nuclear membrane, and prominent nucleoli. In places, fibrillary fibrous stroma fragments are also seen. On magnetic resonance imaging, an approximately 2 x 2 cm heterogeneous enhancing lesion on T1W images was seen in the left cheek infiltrating masseter muscle medially, subcutaneous fat and skin laterally, and abutting anterior border of the left parotid gland posteriorly (Figure 2). She underwent wide local excision after a multidisciplinary tumor board discussion. Intraoperatively, mass was found to be infiltrating the skin, subcutaneous tissue, buccal branch of the facial nerve, anterior border of the parotid gland, and superficial fibers of the masseter muscle. The frozen section of the buccal nerve and parotid tissue was positive for malignancy, hence revised. Postoperative histopathological examination (H&E stain) shows a trabecular and tubular pattern with central ductal cells and abluminal basal cells which favors the diagnosis of basal cell adenocarcinoma.

Surgical steps

**Figure 3:**
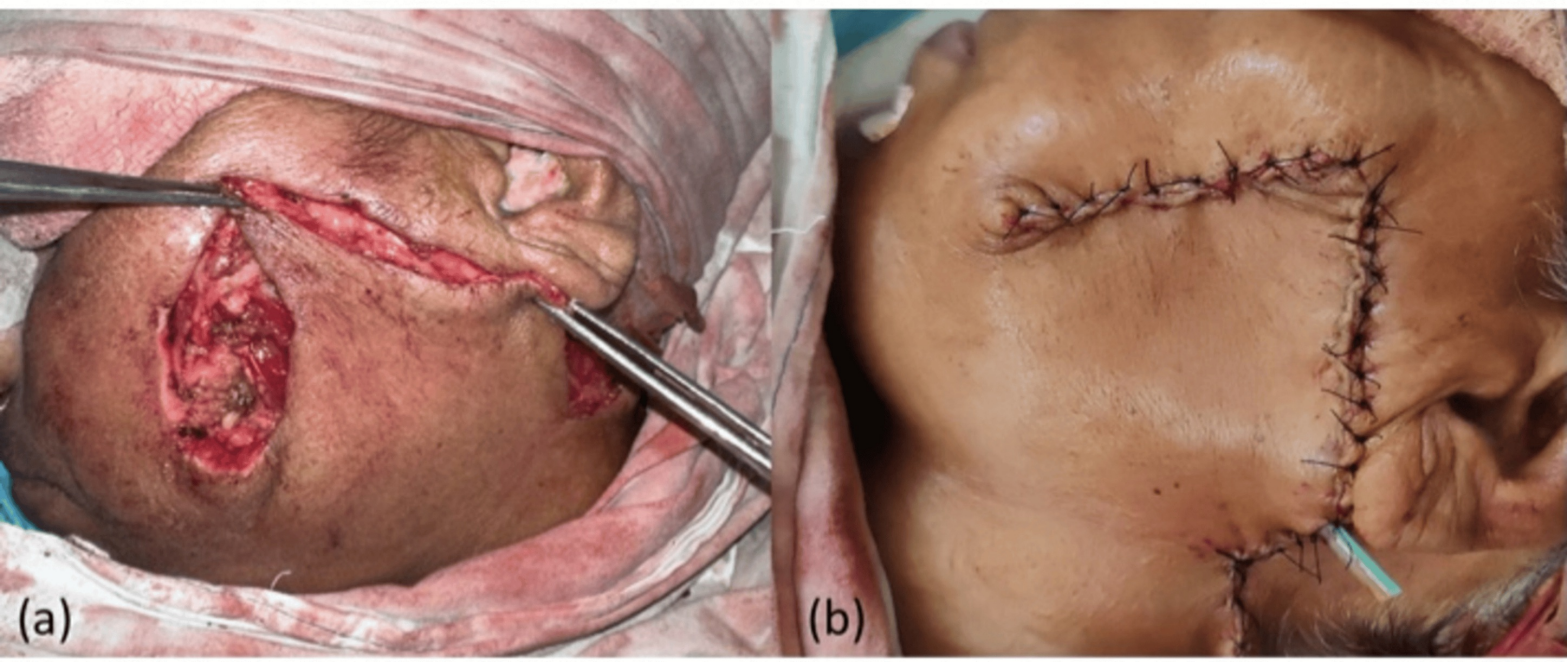
(a) Flap rotated into the defect; (b) final closure of the defect

An incision was made around the swelling in the cheek region of the left side taking margins of approximately 1 cm all around and the flap was elevated as shown in Figure 1a. The tumor was locally excised keeping a margin of 1 cm all around (Figure 1b). The buccal branch of the left facial nerve was infiltrated by the tumor and hence sacrificed (Figure 1c). An inverted U-shaped extension was given to the incision from the pre-auricular area to below the mandible. The cervicofacial flap was raised (Figure 1d) and the defect was sealed by rotating the flap into the defect of the cheek skin as shown in Figure 3a. Hemostasis was achieved and a corrugated rubber drain was placed (Figure 3b). Compression dressing was done after closing the incision in layers.

Histopathology

**Figure 4:**
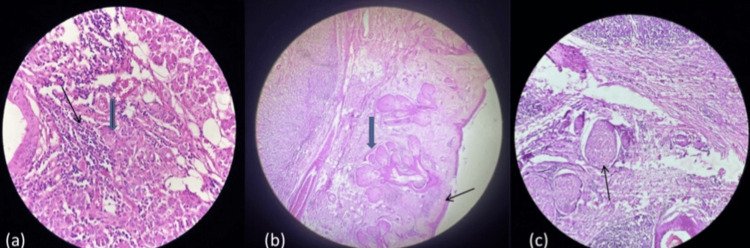
(a) H&E section at 40x shows trabeculae and tubular patterns with central ductal cells (blue block arrow) and abluminal basal cells (black arrow); (b) H&E stained section at 10x shows stratified squamous epithelium (black arrow) and the presence of a salivary gland beneath the epithelium (blue block arrow); (c) H&E section at 40x shows perineural invasion (black arrow)

On histopathological examination, the H&E stained section at 40x magnification shows a trabecular and tubular pattern with central ductal cells and abluminal basal cells. Ductal cells are cuboidal with a moderate amount of cytoplasm and round to oval nucleus while basal cells show scanty cytoplasm with a hyperchromatic ovoid nucleus (Figure 4a). The H&E section at 10x shows stratified squamous epithelium and the presence of a salivary gland beneath the epithelium as in Figure 4b. The H&E section at 40x shows perineural invasion as seen in Figure 4c.

## Discussion

Basal cell adenocarcinoma of the salivary gland is a rare clinical entity that most commonly arises from the parotid gland. Cytological diagnosis of this tumor is exceedingly difficult due to morphologic overlap with other more common biphasic neoplasms of salivary gland and non-salivary gland origin [2]. Though basal cell adenocarcinoma presents as a painless, gradual enlarging swelling in the region of the salivary gland, in our patient it appeared as a nodular skin swelling, which is an atypical presentation. The possible reason for this presentation is that, after originating from the anterior border of the parotid gland, it has grown anteriorly and laterally, sparing the rest of the parotid parenchyma entirely.

Many of the salivary gland, skin adnexal, and cutaneous neoplasms present as a painless slow-growing mass. Clinical presentation, cytology, and even histopathology of these neoplasms can mimic other conditions that should be included in the differential diagnosis [3,4]. Multifocal or membranous type of basal cell adenoma can simulate invasion and this makes differentiation from basal cell adenocarcinoma very difficult. Immunohistochemistry does not play a significant role in the differentiation of basal cell adenoma from adenocarcinoma; however, adequate sectioning including the capsule of the surgical specimen has been suggested [5]. MYB expression has been seen in basal cell adenoma, basal cell adenocarcinoma, and adenoid cystic carcinoma [6]. Basal cell adenocarcinoma can be differentiated from pleomorphic adenoma as it has an abrupt border with the matrix. Canalicular adenomas almost always involve minor salivary glands. Similarly, polymorphous adenocarcinoma occurs in minor salivary glands only. Cutaneous basal cell carcinoma can rarely metastasize to intraparotid lymph nodes or directly infiltrate the parotid gland. It shows basaloid architecture and palisading; however, it lacks a biphasic appearance. Adamantinoma-like Ewing sarcoma is a rare tumor of the head and neck region that can mimic basaloid neoplasm and frequently demonstrates EWSR1-FLI1 translocation [7,8].

Treatment of basal cell adenocarcinoma involves surgery followed by adjuvant therapy (radiotherapy in most instances) in select cases [9]. Parotid gland basal cell adenocarcinoma requires at least a superficial parotidectomy; however, considering the clinical presentation of this patient (since a safe margin could be achieved in parotid tissue), the role of less than superficial parotidectomy can be explored for this low-grade carcinoma. Modifications to the European Salivary Gland Society Classification System for parotidectomy may be useful in this context [10]. In view of the lack of evidence in favor of less than superficial parotidectomy in basal cell adenocarcinoma, the patient was advised to undergo a second surgery, but she refused. She received postoperative radiotherapy considering T4a disease and the presence of perineural invasion.

## Conclusions

The presence of immature cells that resemble cells of the basal layer of epithelium in basaloid neoplasms can easily be misinterpreted. Moreover, the presence of these cells in a wide variety of pathologies further makes the diagnosis difficult. In addition to these problems, this patient presented with basal cell adenocarcinoma, which has grown anteriorly after arising from the anteriormost margin of the left parotid gland. This led to a misdiagnosis based on clinical examination and imaging. Therefore, it is prudent to include all possible pathologies in the differential diagnosis, and the same should be explained to the patient preoperatively.
